# Substance abuse prevention in Cape Town's peri-urban settlements: local health trainers' perspectives

**DOI:** 10.1080/21642850.2013.878659

**Published:** 2014-02-20

**Authors:** Cheneal Puljević, Despina Learmonth

**Affiliations:** ^a^ Psychology Department, University of Cape Town, PD Hahn Building, Cape Town, South Africa

**Keywords:** alcohol abuse, prevention, substance abuse, community health psychology, health trainers

## Abstract

South Africa currently experiences high levels of alcohol and other drug (AOD) abuse. As a result there is a need for the initiation of regional AOD abuse prevention programmes with a specific focus on youth prevention strategies. The Medical Knowledge Institute (MKI) is a non-profit organisation which develops and facilitates health information workshops to members of disadvantaged peri-urban communities in South Africa. This research investigated the views of eight local MKI health trainers on factors contributing to AOD abuse in their communities. Although the expected focus of the discussion was on prevention strategies and effective interventions, the trainers placed more emphasis on the individual and community factors influencing AOD abuse. The themes which emerged through the research included: status, government, (di)stress, gender, recreation, consequences and community. This research holds significance as it has the potential to assist further development of community-based AOD prevention workshops and to guide public health policy and service development for AOD abuse.

## 1. Introduction

### 1.1. Alcohol abuse in South Africa

According to the World Health Organization (WHO), South Africa rates 47th highest out of 189 countries in terms of alcohol consumption, consuming 7.81 litres of absolute alcohol per adult per year (WHO, 2004). However, due to the high numbers of South Africans who abstain from alcohol use, and the high estimated levels of unrecorded and illegal drinking, such as the home-brewing of beer, the real figure is thought to be closer to 16.6 litres per adult (Freeman & Parry, 2006; Schneider, Norman, Parry, Bradshaw, & Plüddemann, 2007). According to Rehm et al. (2003), this is the highest rate of alcohol consumption in the world. In the Western Cape specifically, alcohol remains the most frequently abused substance (Myers, Louw, & Fakier, 2007), with the highest prevalence rates for lifetime alcohol use (39–64%) and risky drinking (9–34%) (Harker et al., 2008).

The general South African alcohol consumption pattern is that of binge and hazardous drinking associated with high levels of related harms (Pasche & Myers, 2012). South Africa has one of the highest rates of foetal alcohol syndrome in the world (May et al., 2007); and levels of alcohol-related injuries reported in trauma centres across South Africa are elevated compared to global figures: estimates suggest that between 36% and 79% of South African trauma patients test positive for alcohol (Plüddemann, Parry, Donson, & Sukhai, 2004). A study conducted in eight police stations across South Africa found that between 6% and 23% of arrestees reported being under the influence of alcohol at the time of committing a crime (Parry, Plüddemann, Louw, & Leggett, 2004).

Alcohol abuse is also associated with increased sexual risk behaviours, for example, unprotected sex and sex with multiple partners (Morojele et al., 2004). These risky sexual behaviours can impact the acquisition and course of HIV. Decreased adherence to anti-retroviral medications is related to regular abuse of alcohol (Hahn, Woolf-King, & Muyindike, 2011). South Africa has amongst the highest rates for HIV infection in the world with approximately 10% of the population known to be infected with HIV (Shisana et al., 2009). As such, it is imperative that all risk factors associated with the spread of HIV, and its management, are addressed.

Alcohol and drug abuse have also been linked to numerous adverse health conditions including heart disease, cancer, Korsakoff's syndrome, strokes and seizures.

Furthermore, the economic costs associated with such widespread abuse of alcohol are estimated to be equal to 1% of South Africa's gross domestic product (over R9 billion per year; Parry, Myers, & Thiede, 2003).

### 1.2. Youth alcohol abuse in South Africa

A recent survey by the South African Medical Research Council's Alcohol and Drug Abuse Research Unit (2013) found that 66% of high-school learners have already used alcohol.

Further surveys also report that more than a third of high-school male students in Cape Town, and over half of male students in Durban regularly engaged in binge-drinking episodes (Parry, Myers, et al., 2004). Heavy drinking by adolescents is associated with absenteeism, academic failure and risky sexual behaviour (Flisher, Parry, Evans, Muller, & Lombard, 2003), as well as an increased likelihood of other illicit drug use (Brook, Morojele, Pahl, & Brook, 2006). Alcohol and other drug (AOD) abuse among adolescents is also linked to an increased risk for suicidal ideation and behaviour (Stoelb & Chiriboga, 1998) and involvement in juvenile crime (Zhang & Wieczorek, 1997).

### 1.3. Illicit drug use in South Africa

Data on illicit drug use in South Africa are relatively less available (Pasche & Myers, 2012). Some recent omnibus surveys have provided some data: the South African Demographic and Health Survey (Department of Health, Medical Research Council, & OrcMacro, 2007), the South Africa National HIV Survey (Shisana et al., 2005), the Youth Risk Behaviour Survey (Reddy et al., 2010), and the South African Community Epidemiology Network on Drug Use (Dada et al., 2011). However, the infrequency of these surveys means that they cannot provide accurate figures. Under-reporting of drug use is also a problem (Pasche & Myers, 2012).

Cannabis (locally termed “dagga”) is the most commonly used illicit drug in South Africa. Cannabis is used by approximately 2% of the population, followed by cocaine (0.3%), sedatives (0.3%), amphetamines (0.2%) and inhalants, hallucinogens and opiates (0.1% each) (Pasche & Myers, 2012; Shisana et al., 2005). However, these South African figures are believed to be grossly underestimated (Pasche & Myers, 2012). In fact, the International Narcotics Board (INCB, 2010) estimates that 15% of the South African population use illegal drugs regularly; this is twice the global norm.

In the Western Cape, the majority of treatment demand is for methamphetamine use (locally referred to as “tik”; Dada et al., 2011). The “Cape Flats” region^1^ in the Western Cape is characterised by a prominent gang culture, associated with a strong drug trade and heavy methamphetamine use (Pasche & Myers, 2012). Currently, approximately 35% of those in treatment in the Western Cape report methamphetamine as their drug of choice (Plüddemann et al., 2010); although again, the real figure is probably much higher.

### 1.4. AOD prevention strategies

Although South Africa arguably has the most developed substance abuse treatment system in Africa, demand for treatment far exceeds supply (Myers, Harker, Fakier, Kader, & Mazok, 2008). Furthermore, affordable state-sponsored treatment facilities have long waiting lists (Myers et al., 2008) and private centres with high standards of care are unaffordable for the majority of the South African population (Pasche & Myers, 2012). Basic logistical barriers also exist for the average South African person in terms of accessing treatment services: competing financial priorities, travel costs and the lengthy travel distances to the nearest service (Myers et al., 2008). These facts, combined with a growing and robust base of scientific evidence for the effectiveness of prevention strategies for AOD abuse (Beardslee, Chien, & Bell, 2011), point to prevention, rather than treatment, as the best possible strategy for addressing the burden of AOD abuse in South Africa.

Research shows that the most effective prevention strategies or interventions for reducing the burden of alcohol abuse to individuals and society is a multi-focused one (Parry, 2005; Parry & Bennetts, 1998). The WHO recommends “a mix of individual- and population-based approaches that target high risk groups and reduce per capita consumption in general” (Parry, 2005, p. 21). Babor et al. (2003), in a study funded by the WHO, reviewed 32 alcohol prevention strategies frequently used by international policy-makers to tackle the burden of alcohol on individuals and society. These strategies were divided into seven categories: regulating physical availability (8 strategies), taxation and pricing (1), altering the drinking context (6), education and persuasion (4), regulating alcohol promotion (2), drinking and driving countermeasures (7), and treatment and early intervention (4) (Parry, 2005). Authors then identified five dimensions common to the most effective strategies: evidence of effectiveness, strength of research support, cross-cultural testing, cost of implementation and target audience (Parry, 2005).

There are a number of specific concerns raised regarding the efficacy of prevention interventions in South Africa (Burnhams, Myers, & Parry, 2009; Parry, 2005). These concerns arise largely due to the fact that there is no national regulating legislation to oversee the training, qualifications and competencies of prevention service providers. There are also no minimum norms or standards to serve as guides for any prevention interventions (Burnhams et al., 2009). As a result of this, not only could the quality of these interventions be compromised, but there is no way of ensuring that prevention services are aware of the latest developments in prevention sciences (Merrill, Pinsky, Killeya-Jones, Sloboda, & Dilascio, 2006). In conjunction with this lack of an organised regulating framework, there is a paucity of research on the overall content and quality of AOD prevention services in South Africa. Consequently, the majority, if not all, organisations rendering prevention services are doing so without knowledge of their effectiveness (Burnhams et al., 2009).

Ten alcohol abuse prevention strategies, identified for their effectiveness in preventing AOD abuse (rating highly on the above five dimensions), are specifically recognised as feasible for implementation within a South African context (Parry, 2005). These include changing the minimum legal purchase age for alcohol (from 18 to 21 years of age), a government monopoly in alcohol retail sales, instituting restrictions on the hours or days of sale, a restriction on the density of outlets, alcohol taxation, an increase in excise taxes on alcohol, sobriety checkpoints, lower blood alcohol concentration limits, administrative licence suspension, graduated licensing for novice drivers and brief interventions for hazardous drinkers.

One policy, however, with no demonstrated empirical efficacy, is the policy of education and persuasion strategies, such as the provision of AOD abuse education in schools and public information campaigns (Parry, 2005). Methods recognised as enhancing the effectiveness of school-based education and persuasion programmes include initiation at primary school level, involvement of parents and the community, peer education, intensive long-term programmes, incorporation of life skills, resistance training and normative concepts, whilst ensuring developmental, cultural and local relevance (Parry, 2005). Nation et al. (2003) identified further characteristics linked to effective AOD abuse prevention interventions. Most overlap with those identified by Parry (2005) with the exception of comprehensive and varied teaching methods delivered by well-trained staff (Nation et al., 2003). Burnhams et al. (2009) also recognised other specific characteristics important to effective prevention programmes, including the importance of a long-term prevention programme (not a once-off intervention, as many South Africa organisations are forced to do), the use of culturally sensitive methods, and the incorporation of AOD prevention education with other life skills training that empowers individuals to make healthy choices and avoid the use of AODs. Effective interventions need to focus on reducing risk factors for and increasing protective factors. In addition, due to the high levels of gang activity in Cape Town's townships (Wilkinson, 2005), these programmes need to address membership of deviant peer groups, learning difficulties or poor school performance and other mental health problems (Burnhams et al., 2009).

The Medical Knowledge Institute (MKI, recently renamed the *Friends of the Health Trainers*) is an international non-profit health-care organisation focused on providing health education and information to disadvantaged communities in developing countries. In Cape Town MKI employs a number of community-based trainers to provide health education and information workshops to disadvantaged populations in Cape Town. One of their training workshops aims to provide education and information on AOD abuse, specifically to youth.

## 2. Aims

MKI approached staff of the Department of Psychology, University of Cape Town, to assist in improving their AOD training programmes. In order to accomplish this, awareness of the opinions, ideas and thoughts of the current trainers was vital. This research study was aimed to investigate the MKI trainers' current perspectives on the current state of AOD abuse in disadvantaged peri-urban communities in Cape Town, including a specific focus on AOD abuse information provision and effective features of AOD prevention strategies.

The investigation of the trainers' views increased our understanding of local barriers to, and facilitators of, AOD abuse and prevention. This enabled better informed and appropriate suggestions for the improvement of MKI's current AOD abuse prevention programme. Ultimately, the goal was, using a combination of the research results with relevant information found in the literature, to create a more generic and *effective* AOD abuse prevention programme that could be introduced to any peri-urban youth population in Cape Town, with successful outcomes.

## 3. Method

### 3.1. Participants

All of the eight trainers employed by MKI were included in the focus groups. The CEO of MKI South Africa also participated in one of the sessions. There were no age, race or gender exclusion criteria. Importantly, all of the trainers currently live in disadvantaged areas in Cape Town. They have been instrumental in the creation and facilitation of AOD abuse workshops for MKI in these areas.

### 3.2. Ethical considerations

This research was approved by the Department of Psychology's Ethics Committee at the University of Cape Town and it adhered to the University of Cape Town's guidelines for research with human subjects. Additionally, the research met the ethical requirements specified by the Research Ethics Department of the Department of Psychology, as well as the Professional Board for Psychology under the Health Professions Council of South Africa (HPCSA, 2002). Participants signed informed consent forms informing them of the confidential, voluntary and anonymous nature of the focus groups. Participants received no financial compensation for their participation. Data recordings were deleted after transcription and participants' names were replaced with letters in order to preserve confidentiality. Transcripts were stored on a password-protected computer.

### 3.3. Data collection

Focus groups, conducted by the authors, were used for data collection. A focus group is generally understood to be a group of participants engaging in group discussion on a particular topic. The conversation is moderated by an interviewer or researcher (Barbour & Kitzinger, 1999).

### 3.4. Procedure

The authors attended two of the monthly trainer feedback meetings in Khayelitsha and Masiphumelele (two peri-urban settlements on the outskirts of Cape Town). The authors were granted permission to attend these meetings by the CEO of MKI South Africa. After obtaining informed consent from individual participants, trainers' discussions were recorded using a voice recorder. These focus groups were semi-structured and focused on the trainers' perceptions of AOD abuse prevention and their opinions on the features of an effective AOD abuse prevention intervention. All recordings were later transcribed in order for the interview content to be analysed as text.

### 3.5. Data analysis

The transcribed data were analysed using thematic analysis. Thematic analysis identifies common themes within the data, and allows for these themes to be grouped in a clear and organised manner (Aronson, 1994). The grouping of data into themes assists in the interpretation of the research topic and allows data to be described in a rich and complex detail (Braun & Clarke, 2006).

Six steps of data analysis, as outlined by Braun and Clarke (2006), were followed. The recommended steps do not follow in a linear fashion, but instead are recursive, where movement is back and forth between the steps, as necessary. The six steps include (a) familiarisation with the data (including transcribing and reading), (b) generating initial codes, (c) searching for themes, (d) reviewing themes, (e) defining and naming themes and (f) producing the report.

Themes highlighted within previous literature, such as the incorporation of life skills training, were initially identified. The emergence of further novel themes emphasised our relative passivity, and elevated the importance of the research above our role as researchers in identifying and interpreting the themes (Braun & Clarke, 2006).

### 3.6. Reflexivity

Reflexivity implies an awareness of our role as the researchers within the research process (Willig, 2001). We remained conscious of our own subjectivity (Camic, Rhodes, & Yardley, 2003), our difference (as white, female, university-educated, middle-class first-language English speakers) and its potential impact on all aspects of the research.

This difference may have cast us as “outsiders” impacting upon the information we were privy to. For example, some of the participants seemed reluctant to answer questions at times, whilst others actively “taught” us about their work. Particularly two of the participants were initially reluctant to speak to us as they reported negative previous experiences with “white people”. These “white people” approached their organisation to do some sort of assistive work, but once the data were collected no report back or follow through ever materialised. We were clearly grouped in with these “white people” intensifying the process of rapport building from the outset.

## 4. Results and discussion

The trainers who took part in the focus groups shared their perceptions of the current state of AOD abuse in Cape Town's disadvantaged communities and identified factors that hinder and promote the provision of training on AOD abuse. Although the expected focus of the discussion was on prevention strategies and effective interventions, the trainers placed more emphasis on the individual and community factors influencing AOD abuse.

### 4.1. Factors which may influence AOD abuse in disadvantaged communities

#### 4.1.1. Status

The trainers repeatedly mentioned that many people living in the townships^2^ drink alcohol in order to gain a sense of status. Young people in these townships appear to respect those who drink alcohol, as the people who are seen buying and drinking alcohol usually dress well and seem to have money. This is despite the fact that many who regularly drink are unemployed, turning to crime or qualifying for social grants in order to generate income.

F: the ones who drink, they will buy the very expensive clothes, they are wearing the Guess jeans and the Carvelas^3^ … because if you wear that name brand stuff and you drink, then you are cool.

In Masiphumelele, officers from the nearby naval base often visit the area over weekends to drink in the *shebeens*.^4^ Young people in Masiphumelele are regularly exposed to these “well-dressed” “desirable” men spending money on alcohol whilst surrounded by attractive women. Research in the Eastern Cape has shown that a significant element of popular African manhood is heterosexual achievement and this is proved in part by being able to attract desirable women (Wood & Jewkes, 2001). This ability improves one's status and asserts masculinity.

The link between money and alcohol also emerged from the data. In fact, in the peri-urban settlements individuals buy particularly expensive brands of alcohol to further enforce the representation of wealth and enhance status; even the colour of beer bottles is associated with wealth and status: somebody drinking beer out of a brown bottle (for example, Black Label, the cheapest beer in South Africa) is considered of a lesser social standing than someone drinking green-bottled drinks (such as Heineken, a slightly more expensive beer).

Likewise, celebrities often act as role models and have an impact on young peoples' perceptions of alcohol and alcohol use. The trainers mentioned how some popular celebrities endorse alcohol brands – a mention was made of celebrity Usher's endorsement of Belvedere Vodka – enhancing the brand's desirability.

As a result of ongoing conditions of racial and economic inequalities, trainers suggested that many poor black people in South Africa believe that they “aren't good enough”. Drinking thus becomes a means of gaining some sense of status in a context of general low self-esteem. This theme links with the literature on absolute deprivation versus relative deprivation which explores the idea that social inequality is far more complex than simple inequality of income (Campbell & Jovchelovitch, 2000). This inequality inevitably leads to poorer health outcomes overall (Wilkinson, 1996, 2005).

#### 4.1.2. (Di)stress

Participants felt that many people living in these townships drink in order to attempt to alleviate stress (including post-traumatic stress) and to try to forget their difficulties, particularly of poverty and exposure to high levels of violence.

F: Many people, they are using drugs and alcohol to reduce stress, and we know exactly where that stress is coming from, we know why they are feeling the way they do, we know why they are so hopeless, we know how much the economy on its own is placing a huge burden.

L: You see men standing there on the street waiting for jobs. These people, they have no home, no job, so when they drink that feeling disappears.

F: It's hard to tell someone not to drink when they come to you and say I was raped by six men and this is the only way I can forget and make people look at me with respect, it is the only way I can feel good enough.

The emergence of this theme supports the earlier notion of stress being a direct cause of increased AOD abuse (Setlalentoa, Thekisho, Ryke, & Loots Du, 2010) The South African population is at high risk for trauma (Carey, Stein, Zungu-Dirwayi, & Seedat, 2003; Williams et al., 2007). Traumatic experiences often result in symptoms of post-traumatic stress disorder (PTSD) and PTSD has been associated with hazardous drinking. In a study of 560 women from a Cape Town township, Watt et al. (2012) found that there was a strong relationship between traumatic events and drinking levels; this was mainly mediated by PTSD symptoms.

Workers from specific industries, such as the brewing and distilling industry, the armed forces and the police, are also more prone to alcohol abuse (Rose-Innes, 2007). These workers engage in risky drinking as a way of coping with social pressure and stress, specifically as a form of “letting off steam” (Setlanlentoa et al., 2010, p. 12).

#### 4.1.3. Government

All of the trainers expressed a desire for the South African government and health-care systems to be more proactive regarding AOD abuse in South Africa.

L: “Even the government do nothing about [AOD abuse].”

Y: I've been talking to our councillor, I told him that we have to do something about this … he said he was going to give me a call but I'm still waiting.

F: We have to have a government who showcases a better lifestyle.

However, when the government occasionally does take action to curb AOD abuse, such as the shutting down of illegal *shebeens* or banning alcohol advertisements, the perception is that the government does not want people to enjoy their lives.

F: The people want to know why is it [that the government takes action to curb AOD abuse] and then the answer will be because they don't want us to enjoy our lives anymore, we drink and enjoy our lives, and it gives the *shebeen* people a boost … and the advertisements, how does that help cutting the advert on TV, that doesn't help.

Modernisation and urbanisation have increased access to, as well as availability of, alcohol and increased exposure to numerous alcohol advertising campaigns (Setlalentoa et al., 2010). Whereas the traditional African style of drinking was limited to special occasions, and to men, in peri-urban settlements and cities drinking is often uninhibited (Jernigan, Monteiro, Room, & Saxena, 2000; Parry & Bennetts, 1998).

#### 4.1.4. Community

The participants pointed to the need for a stronger sense of community in these disadvantaged areas. By increasing the feeling of community, the trainers believed that township residents could take ownership of reducing or preventing AOD abuse.

F: Our community, as much as we are trying to help, they actually have no sense of community, there is no such thing that we are a community. And that's the big problem … Also how we can bring back that sense of community, because it has to start somewhere, that sense of community is quite valuable and important, taking ownership becomes different to action.

L: We need to work together because maybe if we know that guy is selling drugs, we must stand up – work together as a community, and maybe we can call the police because most of the people don't want to. If I know maybe someone is selling drugs, I will just keep quiet for my own safety. Maybe it is time for all of us to stand together to change and help our children and our brothers and sisters.

The trainers also emphasised the need for a community infrastructure to support change linked to the prevention of AOD abuse.

K: You can't just educate someone about [the dangers of AOD abuse] if there is no infrastructure to support it … Change takes time, and the African culture, they don't embrace change easily.

This theme links to the above theme of governmental involvement: the perception that the “government can't fix something they know nothing about”, highlighting the need for the people living in these settlements to take action against AOD abuse themselves.

L: The government, they know nothing about the real situation, they don't know what is actually happening.

F: Because the government does nothing to help, we have a tendency to take the law into our own hands, and try to fix the problem ourselves … We must also be a part of the strategy that [the government] comes up with, because we better understand the challenges, we know what the real problems are.

These sentiments draw attention back to the ideas argued by Wilkinson (1996, 2005), namely, that overall health is superior in more egalitarian societies as a result of the higher social coherence that exists in them.

#### 4.1.5. Gender

Participants spoke about how men cannot refuse an offered alcoholic beverage as this brings their masculinity and sexual orientation into question. In South Africa, heavy alcohol consumption is a practice which flows from hegemonic masculinity; undeniably, social norms around alcohol consumption are one of the reasons why South Africa has one of the highest levels of alcohol consumption per drinker of any country in the world (Coovadia, Jewkes, Barron, Sanders, & McIntyre, 2009; Rehm et al., 2003). Homosexuality is still highly stigmatised in most black African cultures (Campbell, Foulis, Maimane, & Sibiya, 2005; Dlamini, 2006), so practices which may bring a man's masculinity into question, such as abstinence from alcohol, are avoided.

M: Cultures construct what is ok, and as a man you can't say no to a drink or else you're gay. It's like you're not man enough if you say no to a drink.

Women from these peri-urban settlements who visited *shebeens* often had men buy alcoholic drinks for them. Implicit in accepting alcohol from a man was the unspoken agreement that the woman had agreed to sexual relations with that man. Thus, alcohol is believed to be payment for sexual favours and becomes intricately involved in the construction of gender roles. It is not unusual for women and women's sex to be paid for in South Africa. The payment of *lobola* ^5^ by a man for his bride is still widely practised.

Weight loss in young individuals, due to their use of drugs and alcohol, intersects with gender through its attribution to different catalysts. If a young man loses weight, many people assume the young man to be a victim of witchcraft, cursed due to jealousy of his status or success. However, if a young woman loses weight, it is assumed that she has HIV/AIDS. This explanation ties into a general devaluing of women in South Africa. In this context women are frequently stigmatised through representation as the carriers of HIV (Joffe & Begetta, 2003).

The impact of ascriptions of witchcraft or HIV/AIDS on social relations, healthcare and social support for peri-urban South Africans with chronic ill health conditions, such as substance use disorder, is an important factor to consider in terms of prevention and treatment. Incorrect attributions in illness aetiology hinder appropriate prevention and treatment.

#### 4.1.6. Recreation

A specific difficulty mentioned was the lack of safe recreational spaces for the township-based youth. The library is seen as a space for “nerds”, and young people are not allowed to sit near the church “in case they mess the church lawns”. Many peri-urban settlements have not had the advantage of town planning so open spaces for recreation and relaxation generally do not exist. Participants felt that youth are therefore left with few other options than to frequent *shebeens*, where they can play games, such as table tennis or pool, together. In the *shebeens* they are exposed to smoking and drinking, and often encouraged to consume alcoholic beverages.

L: The children are bored, they are just there on the streets.

Trainers also described how many community centres, and the non-governmental organisations running these centres, are forced to focus on HIV prevention in order to obtain necessary funding. As a result, while these spaces once were reserved for activities such as youth dancing groups, they are now used for activities such as gardening for HIV-positive individuals.

A related issue is specifically concerned with the stigma associated with HIV and AIDS. Some of these trainers work in the Desmond Tutu HIV/AIDS Youth Centre in Masiphumelele. Although this is the ideal place for youth to congregate after school and during school holidays, as the centre offers a number of youth-focused activities, many young people are reluctant to spend time there because of the centre's association with HIV and AIDS. The trainers say that youth report that their friends and family asking them “what are they doing at the AIDS centre”. Considering the public stigmatisation of HIV/AIDS and its associated social marginalisation (Campbell et al., 2005), it is not surprising that the local youth do not want to be connected to anything which will engender suspicion around their HIV status.

F: So I'm coming from Desmond Tutu [HIV/AIDS Youth Centre], and I go to a primary school and I go and chat to primary school kids, and they ask me where I work and I tell them I work at the Desmond Tutu centre but I'm working for MKI. They always ask what MKI is, they don't ask what Desmond Tutu is. I try to tell them but because of the assumption, they know it is going to be related HIV, and so they don't care. [HIV/AIDS] is what we as black people are always associated with. We don't want to be associated with that. We are sensitive to such things. And if your mother allows you to go out and she asks you where you were, and you say you were at the Desmond Tutu centre, she'll ask doing what. And even in the household, everyone is asking what you were doing there. They immediately assume that there is a problem.

A similar problem is experienced in Khayalitsha,^6^ as the youth centre there is associated with Baphumelele, an orphanage, which cares for many AIDS orphans.

#### 4.1.7. Consequences

Trainers claimed that the youth are not discouraged from the abuse of drugs and alcohol as they are generally not exposed to individuals experiencing the negative consequences of AOD abuse. In these communities youth often see the drug users when they are “high”: in a good mood and enjoying themselves. They do not see those same users when they are withdrawing or experiencing any other adverse consequences of drug or alcohol abuse. As a result, the youth do not witness the negative effects and so have no tangible disincentives for AOD abuse.

F: It's hard to find people at their most vulnerable moments because they run away from the streets. The only time you see them is when they're high and they're enjoying themselves and they're ignorant and they don't care. And when they are sober, you can't find them anywhere, that's the trickiest thing.

When they do witness someone suffering from the adverse effects of drug use, young people appear to deny the obvious reality as it conflicts with the dominant and more fantastical images of well-dressed desirable men buying and drinking alcohol.

K: They don't see the dirty alcoholics, and if they do, they don't associate the two: the guys drinking alcohol who are well dressed, and that dirty guy lying in the street.

The MKI trainers also reported that that it is difficult to locate individuals who have suffered, or are suffering, the consequences of AOD abuse. As such, the necessity of prevention rather than treatment is once again highlighted.

K: I think the biggest challenge is that we never have the chance to actually speak to the ones who are using drugs or alcohol … Most especially they think [drinking alcohol] is a cool thing to do and they think it's not dangerous as using drugs, so that's the difficult part I think … So all we can do is to try to scare the ones who are not already using alcohol.

The protection motivation theory (PMT) (Rogers, 1975, 1983) examines the cognitive processes through which fear influences persuasion. The PMT posits that people are motivated to protect themselves from physical, psychological and social threats. Response to a threat is based on two cognitive processes: threat appraisal and coping appraisal. A fear appeal will therefore provide impetus for an individual to assess the severity of the event, probability of the event's occurrence and their belief in the efficacy of the message's recommendations. These three factors arouse “protect motivation” which then promotes incentive for change (Keller, 1999). However, if the benefits of the behaviour are believed to outweigh the costs, and individuals possess “optimistic bias”^7^ regarding their personal susceptibility to negative consequences, then they are less likely to respond to fear appeals. As a result, in many instances the use of “scare tactics” may therefore not be effective.

### 4.2. Features of an effective AOD abuse prevention programme for disadvantaged communities

The research findings make it clear that the first focus of a prevention programme should be on the community, as all AOD abuse, and prevention programmes aimed at reducing AOD abuse, must be viewed within context. Campbell and Murray (2004, p. 189) highlight that “a key commitment of community psychologists is to understand what constitutes a ‘health-enabling community context’, and to map out the dialectic of individual and social change involved in promoting such contexts”. They continue to discuss the fact that the challenge in changing health behaviour is not only about changing individual patterns of health, but also contributing to community and social contexts. Positive behaviour change and improvement of personal circumstances are most likely to occur through the concurrent challenge of the social structures that create disadvantages. Collective action increases adoption of health-enhancing behaviours and improves environments for sustained health.

Taking Campbell and Murray's (2004) ideas into account, and acknowledging the trainers' emphasis on the building of a sense of community as a way in which to reduce AOD abuse, AOD interventions need to challenge and address social issues such as ideas around status and alcohol, gender, recreational space and activities, and community responsibility for managing the problem. Parry (2005) has suggested involving parents and the community in AOD abuse prevention programmes, as well as incorporating the element of peer education, which has been identified in the literature as improving the effectiveness of AOD prevention strategies.

Working at an individual level, trainers could perhaps benefit from motivational interviewing skills training. Motivational interviewing enhances individuals' readiness to enact behavioural change (Miller & Rollnick, 2002). If the trainers were familiar with such techniques, they would be more prepared to help AOD workshop participants to promote self-efficacy in reducing their AOD use and supporting health-enhancing environments (Jansen van Vuuren & Learmonth, 2013; Protogerou, Flisher, & Morojele, 2012).

These training workshops should also incorporate life skills training in order to improve their effectiveness (Nation et al., 2003; Parry, 2005). Possible methods of life skills training include: identifying and dealing with emotions, anger and stress management, communication skills, developing assertiveness and peer pressure training.

## 5. Limitations and recommendations

This research explored a small group of community trainers' perceptions of factors which contribute to the high levels of alcohol abuse in South Africa (Setlalentoa, Pisa, Thekisho, Ryke, & Loots, 2010). These trainers commented on their experiences and perceptions of AOD abuse in their specific communities. As a result, these data cannot necessarily be generalised to the wider population. However, insights gained could inform further research into this area, as well as subsequent prevention interventions.

Future recommendations for research studies would include, firstly, the need to create greater community buy-in and establish connections with various important community members and stakeholders before the research commences to enhance the researchers' credibility. Secondly, the use of co-researchers, who are members of the communities and who would be trained as focus group facilitators, could be a solution to the problem of researcher “outsider” status. This may also allow for inclusion of community members in the focus groups, enriching the data through the introduction of different perspectives.

## 6. Conclusion

High levels of AOD abuse in South Africa point to the need for national AOD abuse prevention programmes. Specifically, due to the great risk of South African youth developing AOD problems (Parry, 2005), these AOD prevention strategies should be targeted at South Africa's youth (Burnhams et al., 2009; Parry et al., 2002; Parry, Myers, et al., 2004).

The results of this study give an important insight as to what components contribute to AOD abuse in greater Cape Town's disadvantaged peri-urban communities. Based on these results and a review of the relevant literature, recommendations for the development of effective AOD abuse prevention workshops are suggested. The goal of these workshops should be to dialogue the issues that drive the high levels of AOD abuse, and to focus on community-based solutions to address these issues.

As many of the emergent research factors had social foundations specifically affecting those living in South Africa's disadvantaged communities, it is imperative that a greater awareness of the sociopolitical factors determining health outcomes is actively established with those of influence; whilst empowerment of vulnerable communities, so that they may have a greater chance of health in adversity, also requires a steady focus.

## Notes

1. An expansive, low-lying, flat area outside of Cape Town comprised a number of disadvantaged residential areas.

2. The colloquial name for peri-urban settlements in South Africa.

3. Expensive shoes which are gaining popularity amongst people living in underprivileged communities.

4. Illegal drinking establishments often found in the peri-urban settlements.

5. The bride price is paid by the bridegroom's family in cattle or cash to the bride's family shortly before the marriage.

6. Khayelitsha is a peri-urban township in the Western Cape, located on the Cape Flats in the City of Cape Town. The name is Xhosa for *New Home*. It is reputed to be the largest and fastest growing township in South Africa with a population of approximately 1.2 million.

7. Optimistic bias causes a person to believe that they are less at risk of experiencing a negative event than others.

## References

[CIT0001] Aronson J. (1994). A pragmatic view of thematic analysis. *The Qualitative Report*.

[CIT0002] Babor T., Caetano R., Casswell S., Edwards G., Giesbrecht N., Graham K., Rossow I. (2003). *Alcohol: No ordinary commodity: Research and public policy*.

[CIT0003] Barbour R. S., Kitzinger J. (1999). *Developing focus group research: Politics, theory and practice*.

[CIT0004] Beardslee W. R., Chien P. L., Bell C. C. (2011). Prevention of mental disorders, substance abuse, and problem behaviours: A developmental perspective. *Psychiatric Services*.

[CIT0005] Braun V., Clarke V. (2006). Using thematic analysis in psychology. *Qualitative Research in Psychology*.

[CIT0006] Brook J. S., Morojele N. K., Pahl T., Brook D. W. (2006). Predictors of drug use among South African adolescents. *Journal of Adolescent Health*.

[CIT0007] Burnhams N., Myers B., Parry C. (2009). To what extent do prevention programmes reflect evidenced-based practices? Findings from an audit of alcohol and other drug prevention programmes in Cape Town, South Africa. *African Journal of Drug and Alcohol Studies*.

[CIT0008] Camic P. M., Rhodes J. E., Yardley L. (2003). *Qualitative research in psychology: Expanding perspectives in methodology and design*.

[CIT0009] Campbell C., Foulis C. A., Maimane S., Sibiya Z. (2005). “I have an evil child at my house”: Stigma and HIV/AIDS management in a South African community. *American Journal of Public Health*.

[CIT0010] Campbell C., Jovchelovitch S. (2000). Health, community and development: Towards a social psychology of participation. *Journal of Community and Applied Social Psychology*.

[CIT0011] Campbell C., Murray M. (2004). Community health psychology: Promoting action and analysis for social change. *Journal of Health Psychology*.

[CIT0012] Carey P. D., Stein D. J., Zungu-Dirwayi N., Seedat S. (2003). Trauma and posttraumatic stress in urban Xhosa primary care population: Prevalence, comorbidity and service use patterns. *Journal of Nervous and Mental Disease*.

[CIT0013] Coovadia H., Jewkes R., Barron P., Sanders D., McIntyre D. (2009). The health and health system of South Africa: Historical roots of current public health challenges. *The Lancet*.

[CIT0014] Dada S., Plüddemann A., Parry C. D. H., Bhana A., Vawda M., Fourie D. (2011). http://www.sahealthinfo.org/admodule/sacendu.htm.

[CIT0017] Flisher A. J., Parry C. D. H., Evans J., Muller M., Lombard C. (2003). Substance use by adolescents in Cape Town: Prevalence and correlates. *Journal of Adolescent Health*.

[CIT0018] Freeman M., Parry C. (2006). http://frayintermedia.com/blog/wp-content/uploads/2010/02/Alcohol-Use-Literature-Review.pdf.

[CIT0019] Hahn J. A., Woolf-King S. E., Muyindike W. (2011). Adding fuel to the fire: Alcohol's effect on the HIV epidemic in sub-Saharan Africa. *Current HIV/ AIDS Reports*.

[CIT0020] Harker N., Kader R., Myers B., Fakier N., Parry C., Flisher A. J., Davids A. (2008). *Substance abuse trends in the Western Cape: A review of studies conducted since 2000*.

[CIT0021] Health Professions Council of South Africa. (HPCSA, 2002). http://web.uct.ac.za/depts/psychology/research/Ethics%20Code.doc.

[CIT0022] International Narcotics Control Board. (2010). http://www.incb.org/documents/Publications/AnnualReports/AR2009/AR_09_English.pdf.

[CIT0023] Jansen Van Vuuren A., Learmonth D. (2013). Spirit(ed) away: Preventing foetal alcohol syndrome with MI and CBT. *South African Family Practice*.

[CIT0024] Jernigan D. H., Monteiro M., Room R., Saxena S. (2000). Toward a global alcohol policy: Alcohol, public health and the role of WHO. *Bulletin of the World Health Organisation*.

[CIT0025] Joffe H., Begetta N. (2003). Social representations of AIDS among Zambian adolescents. *Journal of Health Psychology*.

[CIT0027] May P. A., Gossage J. P., Marais A. S., Adnams C. M., Hoyme H. E., Jones K. L., Viljoen D. L. (2007). The epidemiology of fetal alcohol syndrome and partial FAS in a South African community. *Drug Alcohol Dependence*.

[CIT0028] Merrill J. C., Pinsky I., Killeya-Jones L. A., Sloboda Z., Dilascio T. (2006). Substance abuse prevention infrastructure: A survey-based study of the organisational structure and function of the D.A.R.E. program. *Substance Abuse Treatment Prevention Policy*.

[CIT0029] Miller W. R., Rollnick S. (2002). *Motivational interviewing: Preparing people for change.*.

[CIT0030] Morojele N. K., Kachieng'a M. A., Nkoko M. A., Moshia K. M., Mokoko E., Parry C. D. H., Saxena S. (2004). *Perceived effects of alcohol on sexual encounters among adults in South Africa: Dimensions and associations with sexual risk behaviour*.

[CIT0031] Myers B., Harker N., Fakier N., Kader R., Mazok C. (2008).

[CIT0032] Myers B., Louw J., Fakier N. (2007). *Access to substance abuse treatment in the Cape Town Metropole: Final report*.

[CIT0033] Nation M., Crusto C., Wandersman A., Kumpfer K., Seybolt D., Morrissey-Kane E., Davino K. (2003). What works in prevention: Principles of effective prevention programs. *American Psychologist*.

[CIT0034] Parry C. D. H. (2005). South Africa: Alcohol today. Society for the Study of Addiction. *Addiction*.

[CIT0035] Parry C. D. H., Bennetts A. (1998). *Alcohol policy and public health in South Africa*.

[CIT0036] Parry C. D. H., Bhana A., Myers B., Plüddemann A., Siegfried N., Morojele N., Kozel N. (2002). The South African community epidemiology network on drug use (SACENDU): Description, findings (1997–1999), and policy implications. *Addiction*.

[CIT0037] Parry C. D. H., Myers B., Morojele N. K., Flisher A. J., Bhana A., Donson H., Plüddemann A. (2004). Trends in adolescent alcohol and other drug use: findings from three sentinel sites in South Africa (1997–2001. *Journal of Adolescence*.

[CIT0038] Parry C. D. H., Myers B., Thiede M. (2003). The case for an increased tax on alcohol in South Africa. *The South African Journal of Economics*.

[CIT0039] Parry C. D. H., Plüddemann A., Louw A., Leggett T. (2004). The 3-metros study of drugs and crime in South Africa: Findings and policy implications. *American Journal of Drug and Alcohol Abuse*.

[CIT0040] Pasche S., Myers B. (2012). Substance abuse trends in South Africa*.*. *Human Psychopharmacology: Clinical and Experimental*.

[CIT0041] Plüddemann A., Dada S., Parry C., Cerff P., Bhana A., Pereira T., Fourie D. (2010). http://www.sahealthinfo.org/admodule/sacendu.htm.

[CIT0042] Plüddemann A., Parry C., Donson H., Sukhai A. (2004). Alcohol use and trauma in Cape Town, Durban and Port Elizabeth, South Africa: 1999–2001. *Injury Control and Safety Promotion*.

[CIT0044] Reddy S. P., James S., Sewpaul R., Koopman F., Funani N. I., Sifunda S., Omardien R. G. (2010). *Umthente Uhlaba Usamila – the South African youth risk behaviour survey 2008.*.

[CIT0045] Rehm J., Rehn N., Room R., Monteiro M., Gmel G., Jernigan J., Frick U. (2003). The global distribution of average volume of alcohol consumption and patterns of drinking. *European Addiction Research*.

[CIT0046] Rogers R. W. (1975). A protection motivation theory of fear appeals and attitude change. *Journal of Psychology*.

[CIT0049] Schneider M., Norman R., Parry C. D. L., Bradshaw D., Plüddemann A. (2007). *Estimating the burden of alcohol abuse in South Africa in 2000.*.

[CIT0050] Setlalentoa B. M. P., Thekisho G. N., Ryke E. H., Loots Du T. (2010). The social aspects of alcohol abuse/abuse in South Africa. *South African Journal of Clinical Nutrition*.

[CIT0053] Shisana O., Rehle T., Simbayi L. C., Parker W., Zuma K., Bhana A., Piillay V. (2005). *South African national HIV prevalence, HIV incidence, behaviour and communication survey, 2005*.

[CIT0056] Watt M. H., Ranby K. W., Meade C. S., Sikkema K. J., MacFarlane J. C., Skinner D., Kalichman S. C. (2012). Posttraumatic stress disorder symptoms mediate the relationship between traumatic experiences and drinking behavior among women attending alcohol-serving venues in a South African township. *Journal of Studies on Alcohol and Drugs*.

[CIT0057] Wilkinson R. G. (1996). *Unhealthy societies: The afflictions of inequality*.

[CIT0058] Wilkinson R. (2005). *The impact of inequality: How to make sick societies healthier*.

[CIT0059] Williams S. L., Williams D. R., Stein D. J., Seedat S., Jackson P. B., Moomal H. (2007). Multiple traumatic events and psychological distress: The South Africa stress and health study. *Journal of Traumatic Stress*.

[CIT0060] Willig C. (2001). *Introducing qualitative research in psychology. Adventures in theory and method*.

[CIT0061] Wood K., Jewkes R. (2001). *“Dangerous” love: Reflections on violence among Xhosa township youth. Changing men in Southern Africa*.

[CIT0062] World Health Organization. (2004). *WHO global status report on alcohol*.

[CIT0063] Zhang L., Wieczorek W. (1997). The impact of age of onset of substance use on delinquency. *Journal of Research in Crime and Delinquency*.

